# Final Efficacy and Safety Results of Pyrotinib Combined With Trastuzumab and Chemotherapy in Pre‐Treated Human Epidermal Growth Factor Receptor 2‐Positive Metastatic Breast Cancer

**DOI:** 10.1002/cam4.71590

**Published:** 2026-03-15

**Authors:** Xiaofeng Xie, Daijia Huang, Jiayi Huang, Xuelian Chen, Xue Bai, Wenyu Zheng, Liping Chen, Xiaofeng Lan, Lin Song, Rongmei Lei, Caiwen Du

**Affiliations:** ^1^ Department of Medical Oncology National Cancer Center/National Clinical Research Center for Cancer/Cancer Hospital & Shenzhen Hospital, Chinese Academy of Medical Sciences and Peking Union Medical College Shenzhen P.R. China

**Keywords:** metastatic breast cancer, progression‐free survival, survival analysis, target therapy

## Abstract

**Purpose:**

Findings from our previous study showed that the combination of pyrotinib, trastuzumab, and chemotherapy represents a viable treatment strategy with an acceptable safety profile for heavily pre‐treated human epidermal growth factor receptor 2 (HER2)‐positive metastatic breast cancer (MBC). We present here the final efficacy and safety results of our investigation.

**Methods:**

Patients with HER2‐positive MBC who previously received anti‐HER2 therapies such as trastuzumab, pertuzumab, or lapatinib were treated with a combination of pyrotinib, trastuzumab, and chemotherapy. Progression‐free survival (PFS), predictive factors of PFS, and safety were updated in both the intention‐to‐treat population (ITT) and the subgroup exhibiting brain metastases (Sub‐BM). Overall survival (OS) along with its predictive factors was initially analyzed in both ITT and Sub‐BM.

**Results:**

Forty patients were eligible for this analysis. After a median follow‐up of 46.6 months, 27 deaths occurred and four patients continued treatment with pyrotinib combined with trastuzumab and chemotherapy. The median PFS was 7.5 [95% confidence interval (CI), 4.5–10.5 months] and 9.1 months (95% CI, 0.0–18.9 months) in the ITT and Sub‐BM, respectively. The median OS was 32.2 (95% CI, 21.8–42.6 months) and 32.2 months (95% CI, 18.4–46.0 months) in the ITT and Sub‐BM, respectively. Cox regression analyses revealed that liver or/and lung metastases were significant adverse predictive factors for PFS (*p* = 0.020) in the ITT. No new safety concerns were identified following 28 months of additional follow‐up. Adverse events were similar to those reported at the primary analyses with respect to specificity, incidence, and severity.

**Conclusion:**

Updated analyses pertaining to PFS and safety were generally aligned with data obtained from initial assessments. The OS outcomes further substantiated that the combination of pyrotinib, trastuzumab, and chemotherapy is an alternative therapeutic regimen for managing HER2‐positive MBC with heavy pre‐treatment in certain situations, particularly among those with non‐visceral metastases.

## 1 Introduction

Breast cancer (BC) ranks as the most prevalent cancer and the leading cause of cancer‐related mortality worldwide among women, according to Global Cancer statistics 2022 [1]. The human epidermal growth factor receptor 2 (HER2)‐positive (HER2^+^) subtype, which is characterized by overexpression of HER2 or/and amplification of the coding gene *ERBB2*, is aggressive and associated with poor prognosis [2, 3]. Approximately 30% of patients with early‐stage BC will ultimately progress to metastatic breast cancer (MBC), which has a 5‐year overall survival (OS) rate of only 20% [4]. Over the past several decades, anti‐HER2 targeted therapies have significantly improved the clinical outcome of HER2^+^ BC [5].

Initially, Slamon et al. [6] demonstrated that administration of trastuzumab, an anti‐HER2 monoclonal antibody, in conjunction with chemotherapy markedly prolonged progression‐free survival (PFS) and OS in patients with HER2^+^ MBC compared to chemotherapy alone. This finding underscored the critical importance of anti‐HER2 therapy for this subtype. Subsequently, based on results from the phase III CLEOPATRA clinical trial, the addition of pertuzumab to trastuzumab and docetaxel for the frontline management of HER2^+^ MBC resulted in a significant prolongation of median PFS (18.7 months) and OS (57.1 months) compared to placebo, trastuzumab, and docetaxel, which established pertuzumab combined with trastuzumab and a taxane as the preferred first‐line treatment paradigm for HER2^+^ MBC [7]. Notably, the population studied in CLEOPATRA was minimally pretreated; only approximately 10% of patients had previously received trastuzumab, while 23% had received taxanes during earlier disease stages [8]. This situation contrasts sharply with the current landscape where nearly all patients with metastatic HER2^+^ disease have undergone prior treatment with trastuzumab due to its widespread application. A subsequent phase III PHILA trial demonstrated that the combination of pyrotinib, trastuzumab, and docetaxel was superior to placebo plus trastuzumab and docetaxel by significantly improving median PFS to 24.3 months in patients with previously untreated HER2^+^ MBC, providing an alternative first‐line regimen for this patient population [9]. Furthermore, the HER2CLIMB trial indicated significant improvements in both median PFS and OS among patients with more refractory HER2^+^ MBC who received capecitabine plus trastuzumab and tucatinib compared to those not receiving tucatinib. The evidence presented above suggests that dual HER2 blockade is more effective than single HER2 blockade in metastatic disease [10].

By delivering potent chemotherapy directly to HER2^+^ breast cancer cells, antibody‐drugs conjugates (ADCs) provide hope for patients who have been pre‐treated with dual HER2 blockage. Trastuzumab emtansine (T‐DM1) significantly extended PFS and OS while exhibiting lower toxicity compared to lapatinib plus capecitabine in patients with HER2^+^ MBC previously treated with trastuzumab and a taxane [11]. Furthermore, trastuzumab deruxtecan (T‐DXd) elicited a significantly greater improvement in OS (52.6 months) compared to T‐DM1 among patients with HER2^+^ MBC; it also yielded the longest reported median PFS (29.0 months), thereby establishing T‐DXd as the second‐line standard of care (SOC) [12]. However, it is important to note that not all patients benefit from ADCs. HER2 heterogeneity can result in varying levels of dependence on HER2 as an oncogenic driver and different responses to treatments [13]. A post hoc analysis of the MARIANNE trial revealed that patients with tumors harboring heterogeneous HER2 expression had a numerically shorter median PFS duration than those with homogeneous expression, which illustrated a limitation of highly specific HER2‐targeted agents such as T‐DM1 [14]. Although newer generations of ADCs like T‐DXd possess improved pharmacological properties that may help overcome challenges posed by HER2 heterogeneity, their high cost has restricted their accessibility in certain regions.

Tucatinib combined trastuzumab and capecitabine or T‐DM1 is recommended as the preferred regimen for third‐line SOC. In subsequent lines of treatment, there remains no established standard protocol; patients may receive a tyrosine kinase inhibitor (TKI), such as lapatinib, neratinib, or pyrotinib, in combination with capecitabine, or trastuzumab in conjunction with endocrine therapy for cases of hormone receptor‐positive disease, among other options. Despite advancements in HER2‐targeted therapies, most patients undergoing anti‐HER2 targeted therapy ultimately experience disease progression or mortality due to either de novo or acquired resistance in the metastatic setting [5]. Key anti‐HER2 strategies are currently under investigation in HER2^+^ MBC. These strategies include more potent suppression of the HER2 signaling pathway through dual blockade or the development of more effective anti‐HER2 agents designed to overcome acquired resistance to HER2‐targeted therapies [5, 15]. Moreover, it is noteworthy that a substantial proportion of patients with HER2^+^ MBC do not receive treatment with standard first‐ or second‐line regimens due to economic constraints and limited drug availability.

The paradigm of continuing HER2‐targeted therapy through multiple lines of treatment has been established [13]. A phase III study demonstrated that adding trastuzumab to capecitabine after progression on trastuzumab‐based therapy resulted in significantly improved PFS compared to capecitabine alone [16]. Trastuzumab binds to the extracellular domain of the transmembrane HER2 receptor to induce its internalization and degradation and ultimately suppresses cellular growth and proliferation signaling by inhibiting the associated downstream signaling via the RAS/mitogen‐activated protein kinase (MAPK) and phosphoinositide 3‐kinase (PI3K)/AKT pathways [17]. In contrast, pyrotinib, an orally administered irreversible pan‐ErbB TKI binding to the HER2 intracellular kinase domain and further inhibiting the phosphorylation of tyrosine residues and blocking downstream signaling pathways, has shown robust anti‐tumor activity and a manageable safety profile in both early‐stage and metastatic HER2^+^ BC, whether used alone or in combination with other agents [18, 19, 20]. Dual HER2 inhibition through the combination of trastuzumab and pyrotinib, which integrates an extracellular antibody with an intracellular small molecule, has demonstrated synergistic efficacy in both trastuzumab‐sensitive and primary resistant HER2^+^ breast tumors [21, 22].

We conducted a study to evaluate the efficacy and safety of pyrotinib in combination with trastuzumab and chemotherapy for patients with HER2^+^ MBC who had previously received anti‐HER2 therapies and taxanes between November 1, 2018 and March 37, 2021. The initial analysis was followed up to January 20, 2022 [23]. Herein, we present the final efficacy and safety results following an additional 28 months of follow‐up.

## 2 Methods

### 2.1 Study Design

**FIGURE 1:**
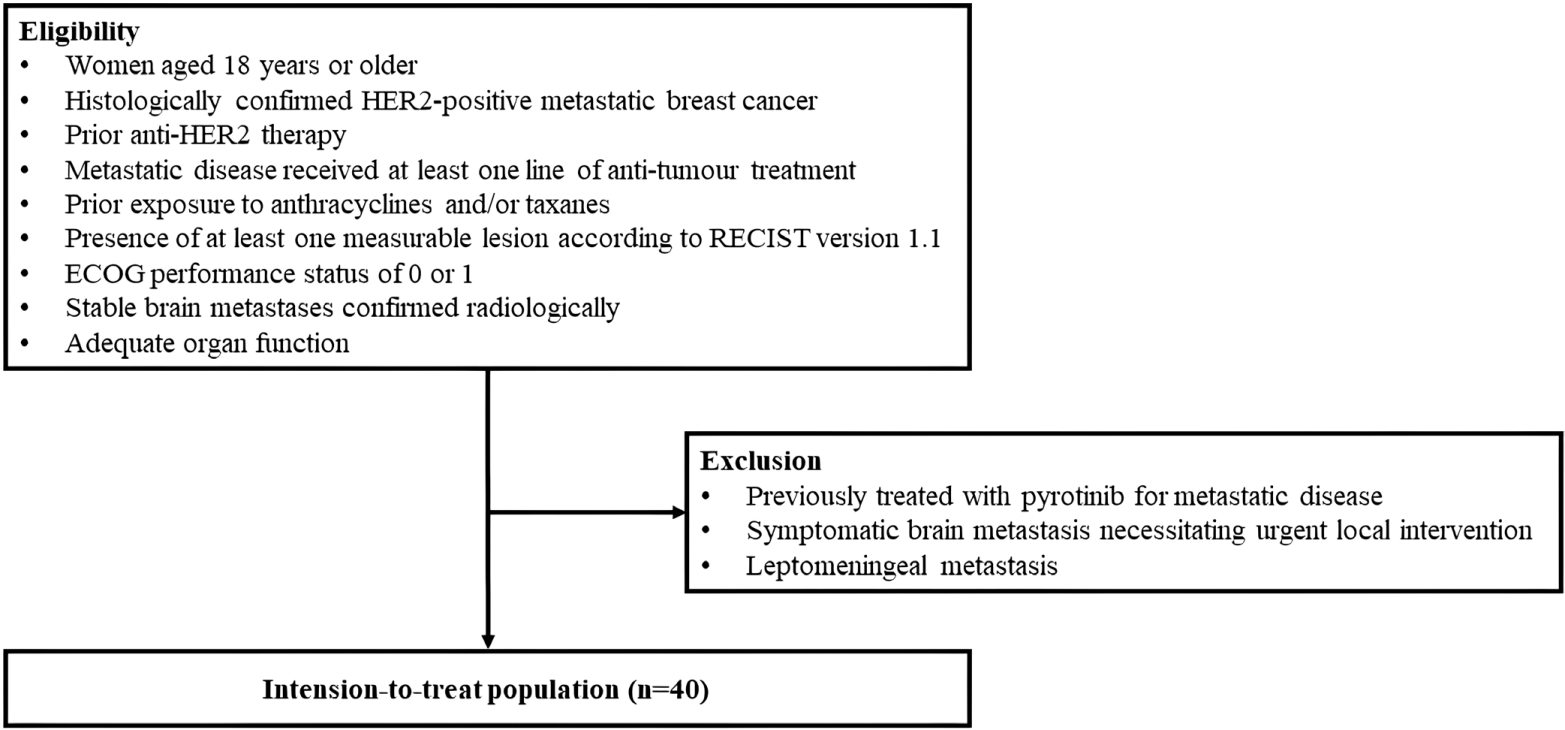
The eligibility and exclusion criteria detailing patient participation in this study. ECOG, Eastern Cooperative Oncology Group; HER2, human epidermal growth factor receptor 2; RECIST, Response Evaluation Criteria in Solid Tumors.

The eligibility and exclusion criteria detailing patient participation in this small sample, single‐arm, exploratory phase II study are presented in Figure 1. The primary endpoint was PFS, defined as the time from treatment initiation to the first documented radiographic evidence of disease progression, death from any cause, or the last follow‐up visit, in the intention‐to‐treat population (ITT). Secondary endpoints were PFS in the subgroup exhibiting brain metastases (Sub‐BM), OS in both ITT and Sub‐BM, objective response rate (ORR), clinical benefit rate (CBR), disease control rate (DCR), safety results, and predictive factors of PFS and OS.

### 2.2 Procedures

Study drugs were administered in 3‐week cycles until disease progression, withdrawal of consent, or occurrence of unacceptable toxic effects. Pyrotinib was administered orally at a dose of 400 mg once daily, with dose reductions (to 320 and 240 mg/day) permitted to manage treatment‐related toxicities. Treatment was discontinued if a pyrotinib dose of 240 mg was not tolerated or if treatment was interrupted for more than 14 days. Trastuzumab was administered intravenously on day 1 of the first cycle at an 8 mg/kg loading dose, changing to a 6 mg/kg maintenance dose for subsequent cycles. The single chemotherapeutic agents were given according to their package inserts. Nab‐paclitaxel was administered intravenously on day 1 at 260 mg/m^2^; capecitabine was given orally twice daily on days 1 to 14 of each 21‐day cycle at 1000 mg/m^2^; gemcitabine was administered intravenously on days 1 and 8 of each 21‐day cycle at 1000 mg/m^2^; vinorelbine was given orally on days 1 and 8 of each 21‐day cycle at 60 mg/m^2^, with an allowance for dose escalation to 80 mg/m^2^ if well tolerated.

### 2.3 Follow‐Up and Assessment

The cut‐off date for this analysis was May 30, 2024, which occurred 28 months after clinical cut‐off for the primary analysis. Throughout the study period, disease response and treatment‐related adverse events (TRAEs) were recorded systematically and in detail. Disease response, primarily assessed through contrast‐enhanced spiral computed tomography and/or contrast‐enhanced magnetic resonance imaging, was evaluated at baseline, every two cycles for the first eight cycles, and every three cycles thereafter in accordance with the Response Evaluation Criteria in Solid Tumors (version 1.1) [24]. TRAEs were monitored continuously and graded according to the National Cancer Institute Common Terminology Criteria for Adverse Events (version 5.0). Performance status, quality of life assessments, and laboratory examinations were assessed at each cycle.

Following cessation of treatment, information regarding survival outcomes and TRAEs was collected via telephone or clinical visits every 9 weeks until death or loss to follow‐up. Each patient was mandatorily contacted to confirm their current survival status prior to the data cut‐offs designated for the PFS and OS analyses.

### 2.4 Statistical Analysis

Statistical analyses were performed using SPSS version 26.0 software (IBM Corporation, Armonk, NY, USA) and GraphPad Prism version 9.0 software (GraphPad Inc., San Diego, CA, USA). The interactions between metastatic sites and prior treatments were assessed using chi‐square or Fisher's exact tests. Kaplan–Meier analysis along with log‐rank tests were utilized to estimate PFS and OS alongside their corresponding 95% confidence intervals (CIs) for both ITT and Sub‐BM. Cox regression analyses were conducted to identify predictive factors influencing PFS and OS outcomes. All reported *p* values were two‐sided, and a *p* value of less than 0.05 was considered statistically significant.

## 3 Results

### 3.1 Study Population

Between November 1, 2018 and March 31, 2021, 40 patients were enrolled and included in the data analyses set. The clinical cut‐off for data collection was May 30, 2024, which occurred 28 months after the clinical cut‐off for the primary PFS analysis and 38 months following the enrollment of the last patient. The median follow‐up duration was 46.6 months [interquartile range (IQR) 43.8–49.3 months]. In this study, 27 deaths were recorded and four patients continued treatment with pyrotinib in combination with trastuzumab and chemotherapy. The median age at diagnosis within the ITT was 46 years (range 31–62 years). Among the participants, 21 patients (52.5%) demonstrated positive estrogen receptor (ER) and/or progesterone receptor (PR) status, while 19 patients (47.5%) were negative for both ER and PR. Thirty‐nine patients (97.5%) had received trastuzumab during either neoadjuvant/adjuvant therapy or for metastatic disease management. Six patients (15%) underwent first‐line combination therapy with pertuzumab and trastuzumab. Twenty‐one patients (52.5%) were treated with TKI‐based anti‐HER2 therapy (lapatinib) specifically due to metastatic progression; however, no prior exposure to T‐DM1 or T‐DXd was documented. Thirteen patients (32.5%) received three or more lines of systemic therapy in the metastatic setting. Additionally, 15 patients (37.5%) presented with baseline brain metastases at study entry; meanwhile, pulmonary and/or hepatic metastasis involvement was observed in 27 patients (67.5%). Among the Sub‐BM, seven cases had previously undergone central nervous system (CNS)‐directed locoregional therapies prior to initiating study treatment. In eight cases, the number of brain metastases exceeded three. The baseline clinicopathological characteristics of 40 patients are detailed in Table 1.

**TABLE 1:** Patient clinical pathological characteristics at baseline.

Characteristics	Cases (%)
ECOG	
0	21 (52.5)
1	19 (47.5)
Age at diagnosis	
≤ 35 years	7 (17.5)
> 35 years	33 (82.5)
Location	
Left	27 (67.5)
Right	13 (32.5)
HER2 status	
IHC 3+	32 (80.0)
IHC 2+ and FISH+	8 (20.0)
Hormone‐receptor status	
ER and/or PR positive	21 (52.5)
ER and PR negative	19 (47.5)
Prior anti‐HER2 treatment	
Trastuzumab	39 (97.5)
Pertuzumab	6 (15.0)
Lapatinib	21 (52.5)
ADCs	0 (0.0)
Prior anthracyclines or taxanes treatment	
Yes	39 (97.5)
No	1 (2.5)
Brain metastasis	
Yes	15 (37.5)
No	25 (62.5)
Local therapy for CNS	
Yes	7 (46.7)
No	8 (53.3)
Number of brain metastasis	
≤ 3	7 (46.7)
> 3	8 (53.3)
Liver or/and lung metastases	
Yes	27 (67.5)
No	13 (32.5)
Number of prior treatment line	
≤ 2	27 (67.5)
> 2	13 (32.5)
Combined chemotherapeutic drug	
Vinorelbine	25 (62.5)
Cepecitabine	6 (15.0)
Gemcitabine	4 (10.0)

Abbreviations: ADCs, antibody‐drugs conjugates; CNS, central nervous system; ECOG, Eastern Cooperative Oncology Group; ER, estrogen receptor; FISH, fluorescence in situ hybridization; HER2, human epidermal growth factor receptor 2; IHC, immunohistochemistry; PR, progesterone receptor.

### 3.2 Efficacy

At the end of follow‐up, there were 34 (85.0%) PFS events in the ITT and 13 (86.7%) in the Sub‐BM. Short‐term efficacy assessments, including ORR of 50.0%, CBR of 75.5%, and DCR of 97.5%, were generally consistent with the primary outcomes observed in all patients [23].

**FIGURE 2:**
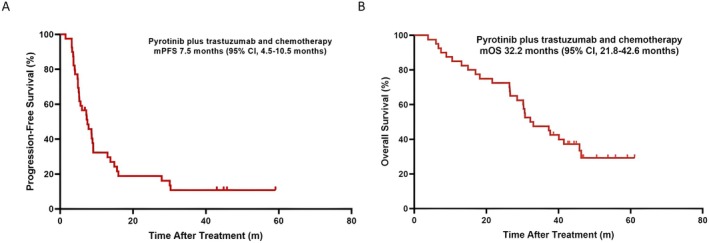
(A) Kaplan–Meier estimates of median progression‐free survival (mPFS) in the intention‐to‐treat population. (B) Kaplan–Meier estimates of median overall survival (mOS) in the intention‐to‐treat population. CI, confidence interval.

**FIGURE 3:**
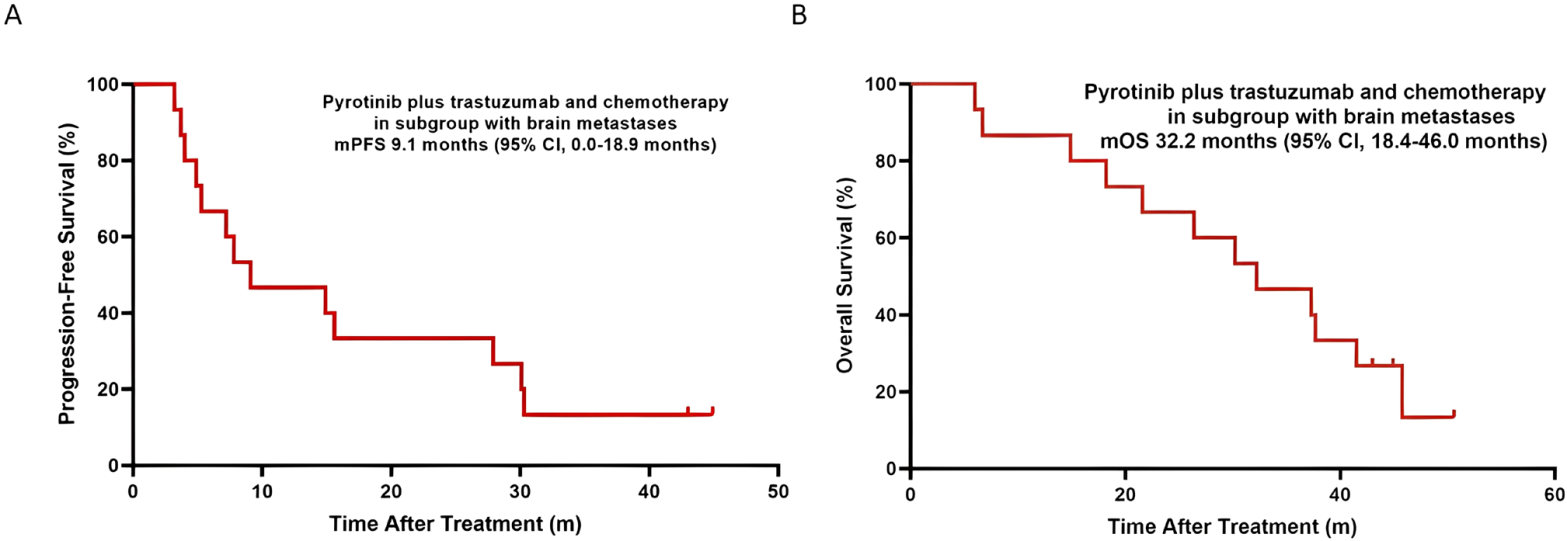
(A) Kaplan–Meier estimates of median progression‐free survival (mPFS) in the subgroup with brain metastases. (B) Kaplan–Meier estimates of median overall survival (mOS) in the subgroup with brain metastases. CI, confidence interval.

The median PFS was determined to be 7.5 months for the ITT (95% CI, 4.5–10.5 months; Figure 2A) and 9.1 months for the Sub‐BM (95% CI, 0.0–18.9 months; Figure 3A). Furthermore, there were 27 (67.5%) OS events in the ITT and 12 (80.0%) in the Sub‐BM. The median OS was 32.2 months each for both ITT (95% CI, 21.8–42.6 months; Figure 2B) and Sub‐BM (95% CI, 18.4–46.0 months; Figure 3B).

**FIGURE 4:**
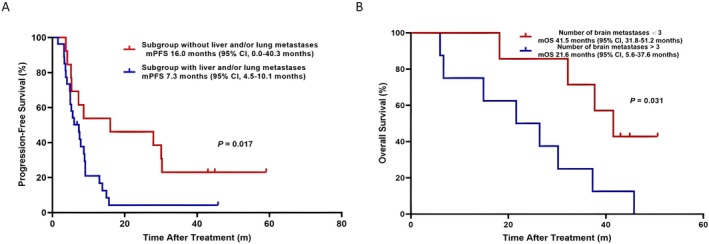
(A) Kaplan–Meier estimates of median progression‐free survival (mPFS) in subgroups with or without liver and/or lung metastases. (B) Kaplan–Meier estimates of median overall survival (mOS) in subgroups categorized by the number of brain metastases (≤ 3 or > 3). CI, confidence interval.

In the ITT, the log‐rank tests indicated that liver or/and lung metastases significantly decreased median PFS; specifically, patients with liver or/and lung metastases experienced a shorter PFS than those without such metastases (7.3 vs. 16.0 months, *p* = 0.017; Figure 4A). In the Sub‐BM, the number of brain metastases was significantly correlated with OS; specifically, patients harboring more than three brain metastases had shorter OS compared to those with fewer than three (21.6 vs. 41.5 months, *p* = 0.031; Figure 4B).

Cox regression analysis was conducted to further identify predictive factors for PFS and OS among the ITT and Sub‐BM. Both univariate and multivariate analyses revealed that the presence of liver or/and lung metastases at baseline served as significant adverse predictors for PFS [hazard ratio (HR) 0.392; 95% CI, 0.177–0.865, *p* = 0.020; Table 2] in the ITT. These findings corroborated results from Kaplan–Meier survival curves and log‐rank tests concerning liver or/and lung metastases' impact on PFS outcomes. However, no significant predictive factors for OS were identified in this analysis (Table 3). Prior anti‐HER2 therapies, including trastuzumab, pertuzumab, lapatinib, and treatments involving anthracyclines or taxanes, were not found to be associated with PFS or OS (Tables 2 and 3). Additionally, no correlation was observed between visceral and brain metastases and prior treatment (Table S1). In the Sub‐BM, although univariate Cox analysis indicated that liver or/and lung metastases, the number of brain metastases, and prior pertuzumab treatment were significant predictors of PFS (HR 0.148; 95% CI, 0.030–0.724, *p* = 0.018; HR 6.830; 95% CI, 1.397–33.387, *p* = 0.018; HR 0.049; 95% CI, 0.004–0.552, *p* = 0.015; Table S2), multivariate Cox analysis suggested that these factors did not serve as independent predictors of PFS (Table S2). Notably, Cox regression analysis indicated that the number of brain metastases was an independent adverse predictor of OS (HR 4.641; 95% CI, 1.050–12.632, *p* = 0.042; Table S3).

**TABLE 2:** Cox regression analysis for PFS of 40 patients with HER2‐positive MBC.

Variables	Univariate analysis			Multivariate analysis		
	HR	95% CI	*p*	HR	95% CI	*p*
Age at diagnosis, year (≤ 60 vs. > 60)	1.244	0.514–3.013	0.629			
Location (left vs. right)	1.566	0.769–3.192	0.217			
HER2 status (IHC 3+ vs. IHC2+ and FISH +)	0.625	0.241–1.625	0.335			
Hormone‐receptor status (ER and/or PR positive vs. ER and PR negative)	0.525	0.261–1.054	0.070			
Prior trastuzumab treatment (yes vs. no)	3.287	0.420–25.697	0.257			
Prior pertuzumab treatment (yes vs. no)	0.724	0.279–1.883	0.508			
Prior lapatinib treatment (yes vs. no)	0.943	0.477–1.864	0.867			
Prior taxanes and/or anthracyclines treatment (yes vs. no)	1.547	0.206–11.618	0.671			
Brain metastases (yes vs. no)	1.595	0.781–3.216	0.202			
Liver or/and lung metastases (yes vs. no)	0.392	0.177–0.865	**0.020**	0.392	0.177–0.865	**0.020**
Number of previous treatment line (≤ 2 vs. > 2)	1.185	0.575–2.440	0.646			
Combined chemotherapeutic drug (Vinorelbine vs. Cepecitabine vs. Nab‐paclitaxel)	1.198	0.855–1.681	0.294			

*Note:* Bold: *p‐value of less than 0.05 was considered statistically significant.

Abbreviations: CI, confidence interval; ER, oestrogen receptor; FISH, fluorescence in situ hybridization; HER2, human epidermal growth factor receptor; HR, hazard ratio; IHC, immunohistochemistry; MBC, metastatic breast cancer; PFS, progression‐free survival; PR, progesterone receptor.

**TABLE 3:** Cox regression analysis for OS of 40 patients with HER2‐positive MBC.

Variables	Univariate analysis			Multivariate analysis		
	HR	95% CI	*p*	HR	95% CI	*p*
Age at diagnosis, years (≤ 60 vs. > 60)	1.496	0.515–4.345	0.459			
Location (left vs. right)	0.814	0.355–1.864	0.626			
HER2 status (IHC 3+ vs. IHC2+ and FISH+)	0.730	0.275–1.937	0.527			
Hormone‐receptor status (ER and/or PR positive vs. ER and PR negative)	1.164	0.546–2.481	0.694			
Prior trastuzumab treatment (yes vs. no)	3.809	4.082–30.087	0.205			
Prior pertuzumab treatment (yes vs. no)	0.988	0.341–2.863	0.982			
Prior lapatinib treatment (yes vs. no)	0.945	0.440–2.029	0.884			
Prior taxanes and/or anthracyclines treatment (yes vs. no)	0.830	0.111–6.181	0.856			
Brain metastases (yes vs. no)	0.649	0.302–1.396	0.269			
Liver or/and lung metastases (yes vs. no)	0.677	0.296–1.549	0.355			
Number of previous treatment line (≤ 2 vs. > 2)	1.373	0.610–3.091	0.444			
Combined chemotherapeutic drug (Vinorelbine vs. Cepecitabine vs. Nab‐paclitaxel)	1.233	0.859–1.768	0.256			

Abbreviations: CI, confidence interval; ER, estrogen receptor; FISH, fluorescence in situ hybridization; HER2, human epidermal growth factor receptor; HR, hazard ratio; IHC, immunohistochemistry; MBC, metastatic breast cancer; OS, overall survival; PR, progesterone receptor.

### 3.3 Safety

All TRAEs in the study reported at the first data cut‐off in January 2022 [23] and after an additional 28 months of follow‐up were similar with respect to incidence, severity, and specificity, which have been updated and summarized in Table 4. During the extended follow‐up period, new occurrences of Grade 1 anemia and Grade 1 diarrhea were documented. Diarrhea, leukopenia, neutropenia, fatigue, vomiting, nausea, hand‐foot syndrome, and aspartate transaminase elevation remained the most frequently reported adverse events, listed in descending order of frequency. As indicated by preliminary analyses, the majority of TRAEs can be effectively managed through dose reduction strategies and supportive care measures.

**TABLE 4:** Treatment‐related adverse events occurring in the intention‐to‐treat population.

Adverse events	Any grade (%)	Grade ≥ 3 (%)
Hematologic		
Leukopenia	17 (42.5)	5 (12.5)
Neutropenia	15 (37.5)	6 (15.0)
Anemia	3 (7.5)	0
Non‐hematologic		
Diarrhea	35 (87.5)	10 (25.0)
Vomiting	13 (32.5)	1 (2.5)
Nausea	5 (12.5)	0
Hand‐foot syndrome	4 (10.0)	0
Aspartate transaminase elevation	4 (10.0)	1 (2.5)
Anorexia	3 (7.5)	0
Alanine aminotransferase elevation	2 (5.0)	0
Peripheral neurotoxicity	2 (5.0)	0
Rash	1 (2.5)	0
Thrombocytopenia	1 (2.5)	1 (2.5)

## 4 Discussion

This final analysis of mature survival data substantiates that the therapeutic efficacy of pyrotinib combined with trastuzumab and chemotherapy remains robust for heavily pre‐treated HER2^+^ MBC patients, even after a median follow‐up duration of 46.6 months. The treatment regimen demonstrated a median PFS of 7.5 months and an ORR of 50% in the ITT, which was generally consistent with previously published data (median PFS: 7.5 months; ORR: 50%) [23]. Importantly, the OS was initially documented at 32.2 months. The median OS reported in this study aligns closely with those observed in the EMILIA trial (30.9 months), which utilized one of the recommended second‐line treatment options.

Dual HER2 blockade through the combination of a TKI and trastuzumab has shown potential to overcome resistance to HER2‐targeted drugs, resulting in favorable therapeutic outcomes [10, 25]. The pivotal phase III HER2CLIMB clinical trial initially established the superior therapeutic efficacy of trastuzumab in combination with TKI tucatinib [10]. Subsequently, the phase III PHILA study reaffirmed the robust efficacy of dual HER2 blockade via pyrotinib combined with trastuzumab [9]. Liu et al. [22] demonstrated that pyrotinib plus trastuzumab exhibits superior inhibition in the HER2 signaling pathway compared to pyrotinib or trastuzumab alone by inducing membrane HER2 ubiquitin‐mediated downregulation, especially in the context of HER2‐dependent BC. Numerically, datasets accumulated from the PHENIX and PHOEBE studies involving pyrotinib‐based regimens exhibited superior quality compared to those obtained in our investigation [26, 27], a finding that may be partially ascribed to differences in patient characteristics including fewer lines of prior treatment and a higher proportion of trastuzumab‐naïve and lapatinib‐naïve patients. In our study, it is noteworthy that 97.5% of the patients had previously received trastuzumab therapy while 52.5% had been treated with lapatinib. This indicates that continuous inhibition of the HER2 signal pathway by combining pyrotinib with trastuzumab may reverse resistance to either trastuzumab or lapatinib. Proposed synergistic mechanisms may involve enhanced apoptosis of cancer cells, increased stabilization and degradation of HER2 receptors, as well as reversion of resistance to trastuzumab by accumulation of HER2 receptors on the surface of BC cells [28, 29]. Additionally, pyrotinib is an irreversible pan‐ErbB TKI that targets HER1, HER2, and HER4, while lapatinib is a reversible small dual TKI specific for HER1 and HER2 [30]. Consequently, the response observed in patients previously treated with lapatinib may be attributed in part to chemotherapeutic agents and in part to continuous inhibition of the HER2 signal pathway resulting from the reversal of resistance to lapatinib achieved by combining pyrotinib and trastuzumab. In the current scenario where T‐DXd has become the standard therapeutic option, the combination of trastuzumab with pyrotinib and chemotherapy offers an alternative treatment regimen for patients in regions where T‐DXd is unavailable or for those who have experienced disease progression following T‐DXd therapy.

Although the survival of patients with HER2^+^ MBC has significantly improved with the introduction of HER2‐target therapies, CNS events continue to pose a formidable challenge and remain a major source of mortality for these patients [31, 32, 33]. In our study, the median PFS was updated to 9.1 months from a previous report of 9.4 months, while the median OS was initially reported as 32.2 months for the Sub‐BM. As previously documented, the median PFS observed in the Sub‐BM demonstrated numerically superior outcomes compared to that in the ITT. This discrepancy is principally attributable to differences in prior treatment exposure, characterized by a higher proportion (32.5% vs. 20.0%) of patients in the ITT having received more than two lines of prior treatment. Furthermore, the median OS in the Sub‐BM numerically approximated that observed in the ITT. In real‐world studies, patients with brain metastases exhibited shorter OS compared to those without such involvement, indicating that BM is a poor factor for clinical outcomes [34]. Nevertheless, advancements in both systemic and local management have also tremendously improved OS among HER2^+^ patients with brain metastases. In the HER2CLIMB trial, which evaluated tucatinib combined with trastuzumab and capecitabine in patients with HER2^+^ MBC and brain metastases, median PFS was recorded at 7.6 months [10]. Numerically, the PFS for Sub‐BM reported in this study was 9.1 months, which exceeds that observed in the HER2CLIMB trial at 7.6 months. The intrinsic efficacy of pyrotinib against intracranial metastases may partly explain this difference. The PERMEATE study, a multi‐center, single‐arm phase II trial that investigated the efficacy and safety of pyrotinib combined with capecitabine in individuals with HER2^+^ MBC accompanied by brain metastases, revealed a median PFS of 11.3 months within the radiotherapy‐ or surgery‐naïve group versus 5.6 months among those treated previously by either modality [35]. Furthermore, it is plausible that local therapeutic interventions for intracranial metastatic lesions—administered to 46.7% of patients with brain metastases—contributed to enhanced blood–brain barrier permeability, thereby facilitating increased intracranial concentrations of systemically administered antineoplastic agents. It should be noted that the HER2CLIMB study represents a randomized controlled phase III clinical trial with a substantial sample size and provides a high level of evidence within evidence‐based medicine; nearly 50% of its enrolled participants had brain metastases. In contrast, the current study represents a limited sample phase II exploratory investigation. Based on existing evidence‐based data, tucatinib has been established as one of the confirmed optimal treatment options for patients with brain metastases. For countries or regions where tucatinib has not yet received approval, pyrotinib may also be considered as a clinically valuable alternative treatment option.

Predictive factors are essential for optimizing the benefits of pyrotinib combined with trastuzumab and chemotherapy in individuals with HER2^+^ MBC. Regarding the impact of metastatic sites on survival outcomes in MBC, it has been established that patients with visceral metastases experience a poorer prognosis compared to those with non‐visceral metastases such as chest wall recurrence or bone/soft tissue metastases, which is further associated with reduced OS [36, 37]. The current results reinforced our previous findings indicating that liver or/and lung metastases served as independent adverse predictors for PFS, suggesting that patients with non‐visceral metastases have significantly longer PFS compared to those presenting with visceral involvement. The administration of pyrotinib in conjunction with chemotherapy within a metastatic context had been documented to yield more favorable tumor responses and extended PFS among patients exhibiting non‐visceral metastases [38]. The findings from our study align fundamentally with existing research, indicating that individuals harboring non‐visceral metastases may derive greater benefit from this combined therapeutic approach. These insights hold potential implications for patient selection and may optimize clinical management strategies.

Approximately 35%–50% of patients with HER2^+^ BC develop brain metastases with poorer outcomes; the median OS typically does not exceed 12 months. Consequently, it is crucial to investigate predictors of PFS and OS in patients with brain metastases based on distribution‐balanced clinicopathological features. No statistically significant independent predictors for PFS were identified in the Sub‐BM. The number of brain metastases was reported as an independent predictor for OS. That is to say, patients receiving trastuzumab combined with pyrotinib and chemotherapy exhibited significantly improved OS when the number of brain metastases was ≤ 3 compared to those with > 3, thereby providing essential evidence for precision treatment selection. Prior studies have established associations between the number of brain metastases and survival outcomes [39]. Additionally, this research demonstrated that a history of local therapy and prior systemic treatment regimens had no significant impact on either PFS or OS. It is important to acknowledge that limitations arising from the current sample size (*n* = 15) necessitate further validation through multicenter prospective studies involving larger samples.

The TRAEs observed in this study were aligned with the established safety profile of pyrotinib in conjunction with trastuzumab and chemotherapy, with no novel TRAEs being documented. The TRAEs reported following an additional 28 months of follow‐up were generally consistent with those identified in the primary analyses regarding incidence, severity, and specificity. Diarrhea, which typically occurs between Days 4 and 14 following the initial dose, remains the most prevalent TRAE and is predominantly induced by pyrotinib. This adverse event can be effectively managed through dose reduction and administration of loperamide, becoming significantly more tolerable 1 month after initiation. Other adverse events reported at a frequency of at least 10%, including leukopenia, neutropenia, fatigue, vomiting, nausea, hand‐foot syndrome, and elevations in aspartate transaminase, were also found to be manageable. Importantly, no cardiac‐related events were observed among patients receiving the combination therapy of pyrotinib and trastuzumab. Following this extended follow‐up period, the manageability of the TRAEs alongside the low incidence of severe adverse events further validates the safety profile of pyrotinib combined with trastuzumab for patients with heavily pre‐treated MBC.

In terms of security, we conducted a horizontal comparison. In the initial analyses of the DESTINY‐Breast 03 trial, the most frequently reported drug‐related TRAEs of any grade in the T‐DXd group were nausea (72.8%), fatigue (44.7%), and vomiting (44.0%) [40]. Subsequently, in the long‐term survival analysis of the DESTINY‐Breast 03 trial, it was noted that 22.6% of patients receiving T‐DXd discontinued treatment due to drug‐related adverse events; among these, pneumonitis (6.6%) and interstitial lung disease (5.4%) were identified as the most common TRAEs associated with discontinuation [12]. Therefore, caution should be exercised regarding long‐term use of T‐DXd with respect to ILD. In HER2CLIMB [10], the predominant adverse events observed among patients receiving a tucatinib‐based regimen included diarrhea, palmar‐plantar erythrodysesthesia syndrome, nausea, fatigue, and vomiting; most cases were classified as grade 1 or 2 and less than 6% of patients discontinued treatment. Noteworthy safety events included diarrhea that was managed with short courses of antidiarrheal agents and transient reversible elevations in liver enzyme levels. Overall, the combination of trastuzumab and TKI has demonstrated favorable long‐term tolerability. However, due to the significant risk of cardiac toxicity associated with prolonged use of trastuzumab [6], careful monitoring for cardiotoxic effects is warranted during long‐term administration.

As previously indicated, this study has several limitations that include its exploratory and unicentric nature, a limited sample size of both ITT and Sub‐BM, and a relatively low rate of standard care administration in previous lines of treatment among the enrolled patients due to historical factors. With the increasing utilization of HER2‐targeted agents, including novel anti‐HER2 antibodies, TKIs, and ADCs, there is a necessity for multi‐center, randomized controlled trials involving larger cohorts to further substantiate the efficacy and safety of this combined regimen in HER2^+^ MBC patients with a history of standard treatment, including those with untreated metastasis. Moreover, while the findings imply that patients devoid of liver or/and lung metastases may derive greater benefit from this regimen, further investigation into the underlying genetic mechanisms is warranted.

## 5 Conclusion

The efficacy and safety of pyrotinib combined with trastuzumab and chemotherapy observed in previous analyses were maintained after a median follow‐up period of nearly 4 years. This offers an alternative active option for heavily pre‐treated HER2^+^ MBC patients, particularly those with non‐visceral metastases.

## Author Contributions

Caiwen Du: conceptualization and design of the study, provided final approval for manuscript publication; Jiayi Huang and Xuelian Chen: literature review; Xue Bai, Wenyu Zheng, Liping Chen, Xiaofeng Lan, Lin Song, and Rongmei Lei: data collection; Xiaofeng Xie and Daijia Huang: data analysis and interpretation as well as drafting and editing of the manuscript. All authors accept full responsibility for the content and have thoroughly reviewed and approved it for submission.

## Funding

This research was financially sponsored by National Cancer Center/National Clinical Research Center for Cancer/Cancer Hospital & Shenzhen Hospital, Chinese Academy of Medical Sciences and Peking Union Medical College, Shenzhen (No. E010322029), Shenzhen Science and Technology Program (2018, JCYJ20180306171227129), Shenzhen Key Medical Discipline Construction Fund (No. SZXK013), and Sanming Project of Medicine in Shenzhen (No. SZSM202411002).

## Ethics Statement

All procedures conducted involving human participants were in accordance with the 1964 Helsinki Declaration and its subsequent amendments or comparable ethical standards. This study was also approved by institutional review boards and ethics committees (approval number: 2019‐35) of the National Cancer Center/National Clinical Research Center for Cancer/Cancer Hospital & Shenzhen Hospital, Chinese Academy of Medical Sciences and Peking Union Medical College (Shenzhen, Guangdong, China).

## Consent

All individual participants provided written informed consent for the use of their medical information for research purposes. The authors affirmed that human research participants provided informed consent for publication.

## Conflicts of Interest

The authors declare no conflicts of interest.

## Supporting information


**Data S1:** cam471590‐sup‐0001‐TableS1‐S3.docx.

## Data Availability

The datasets generated and/or analyzed in the course of the current study are accessible from the corresponding author upon reasonable request.
