# Supplementary material for: Final Efficacy and Safety Results of Pyrotinib Combined With Trastuzumab and Chemotherapy in Pre‐Treated Human Epidermal Growth Factor Receptor 2‐Positive Metastatic Breast Cancer
Source: Cancer Med. 2026 Mar 15;15(3):e71590. doi: 10.1002/cam4.71590 (PMC13093436; doi:10.1002/cam4.71590)
Supplement: Supplementary file 1 — Data S1: cam471590‐sup‐0001‐TableS1‐S3.docx. [file CAM4-15-e71590-s001.docx]

**Supplementary Tables**

**Table S1. Associations between metastatic sites and prior treatment in 40 patients with HER2-positive MBC**

| **Prior treatment** | **Lung and/or liver metastases**  **[cases (%)]** | |  | **Brain metastasis**  **[cases (%)]** | |  | |
| --- | --- | --- | --- | --- | --- | --- | --- |
|  |  |  | ***P* value** |  |  | ***P-*value** | |
|  | **Yes** | **No** |  | **Yes** | **No** |  |  |
| Total | 40 | |  | 40 | |  | |
| Trastuzumab |  |  | 1.000 |  |  | 0.375 | |
| Yes | 26 (96.3) | 13(100.0) |  | 14 (93.3) | 25 (100.0) |  | |
| No | 1 (3.7) | 0 (0.0) |  | 1 (6.7) | 0 (0.0) |  | |
| Pertuzumab | |  | 0.643 |  |  | 1.000 | |
| Yes | 5 (18.5) | 1 (7.7) |  | 2 (13.3) | 4 (16.0) |  | |
| No | 22 (81.5) | 12 (92.3) |  | 13 (86.7) | 21 (84.0) |  | |
| Lapatinib |  |  | 0.738 |  |  | 0.745 | |
| Yes | 15 (55.6) | 6 (46.2) |  | 7 (46.7) | 14 (56.0) |  | |
| No | 12 (44.4) | 7 (53.8) |  | 8 (53.3) | 11 (44.0) |  | |
| Anthracyclines and/or taxanes | |  | 1.000 |  |  | 1.000 | |
| Yes | 26 (96.3) | 13 (100.0) |  | 15 (100.0) | 24 (96.0) |  | |
| No | 1 (3.7) | 0 (0.0) |  | 0 (0.0) | 1 (4.0) |  | |

**HER2,** human epidermal growth factor receptor; **MBC,** metastatic breast cancer

| **Table S2. Cox regression analysis for PFS of 15 patients with Sub-MB** | | | | | | | | |
| --- | --- | --- | --- | --- | --- | --- | --- | --- |
| **Variables** | **Univariate analysis** | | |  | **Multivariate analysis** | | | |
|  | **HR** | **95% CI** | ***P-*value** |  | **HR** | **95% CI** | ***P-*value** | |
| Hormone-receptor status (ER and/or PR positive vs ER and PR negative) | 0.487 | 0.154-1.543 | 0.221 |  |  |  |  | |
| Liver or/and lung metastases (yes vs no) | 0.148 | 0.030-0.724 | **0.018** |  | 0.363 | 0.042-3.125 | 0.356 | |
| Number of previous treatment line (≤ 2 vs > 2) | 1.484 | 0.380-5.795 | 0.570 |  |  |  |  | |
| Number of brain metastasis (≤ 3 vs > 3) | 6.830 | 1.397-33.387 | **0.018** |  | 2.831 | 0.331-24.235 | 0.342 | |
| Prior pertuzumab treatment (yes vs no) | 0.049 | 0.004-0.552 | **0.015** |  | 0.108 | 0.009-1.269 | 0.077 |  |
| Prior lapatinib treatment (yes vs no) | 0.699 | 0.225-2.174 | 0.537 |  |  |  |  |  |
| Prior local treatment for CNS (yes vs no) | 0.839 | 0.275-2.563 | 0.758 |  |  |  |  |  |
| **PFS,** progression-free survival; **Sub-MB,** subgroup exhibiting brain metastases**; HR,** hazard ratio; **CI,** confidence interval; **ER,** oestrogen receptor; **PR,** progesterone receptor; **CNS,** central nervous system. | | | | | | | |  |

| **Table S3. Cox regression analysis for OS of 15 patients with Sub-MB** | | | | | | | |
| --- | --- | --- | --- | --- | --- | --- | --- |
| **Variables** | **Univariate analysis** | | |  | **Multivariate analysis** | | |
|  | **HR** | **95% CI** | ***P-*value** |  | **HR** | **95% CI** | ***P-*value** |
| Hormone-receptor status (ER and/or PR positive vs ER and PR negative) | 1.501 | 0.452-4.983 | 0.507 |  |  |  |  |
| Liver or/and lung metastases (yes vs no) | 0.390 | 0.119-1.282 | 0.121 |  |  |  |  |
| Number of previous treatment line (≤ 2 vs > 2) | 2.855 | 0.628-12.988 | 0.175 |  |  |  |  |
| Number of brain metastasis (≤ 3 vs > 3) | 4.641 | 1.050-12.632 | **0.042** |  |  |  |  |
| Prior pertuzumab treatment (yes vs no) | 0.402 | 0.083-1.955 | 0.259 |  |  |  |  |
| Prior lapatinib treatment (yes vs no) | 1.076 | 0.326-3.549 | 0.904 |  |  |  |  |
| Prior local treatment for CNS (yes vs no) | 0.659 | 0.209-2.079 | 0.477 |  |  |  |  |
| **OS,** overall survival; **Sub-MB,** subgroup exhibiting brain metastases**; HR,** hazard ratio; **CI,** confidence interval; **ER,** oestrogen receptor; **PR,** progesterone receptor; **CNS,** central nervous system. | | | | | | | |
